# Signals of adverse drug reactions communicated by pharmacovigilance stakeholders: protocol for a scoping review of the global literature

**DOI:** 10.1186/s13643-020-01429-z

**Published:** 2020-08-13

**Authors:** Daniele Sartori, Jeffrey K. Aronson, Igho J. Onakpoya

**Affiliations:** 1 Uppsala Monitoring Centre, Bredgränd 7B, 753 20 Uppsala, Sweden; 2 Centre for Evidence-Based Medicine, Nuffield Department of Primary Care Health Sciences, University of Oxford, Woodstock Road, Oxford, OX2 6GG United Kingdom

**Keywords:** Signal, Adverse event, Adverse drug reaction, ADR, Scoping review, Protocol, Signal detection, Pharmacovigilance

## Abstract

**Background:**

Signals of adverse drug reactions (ADRs) form the basis of some regulatory risk-minimization actions in pharmacovigilance. Reviews of limited scope have highlighted that such signals are mostly supported by reports of ADRs or multiple types of evidence. The time that elapses between a report of a suspected ADR and the communication of a signal has not been systematically characterized. Neither has the features of reports of suspected ADRs that authors used to support putative causal relationships, although difficulties with establishing causal relationships between medicinal products and adverse events have been highlighted. The objectives of this study will be to describe the evidence underpinning signals in pharmacovigilance, the features of reports of ADRs supporting signals, and the time that it takes to communicate a signal.

**Methods:**

We shall retrieve records from PubMed, EMBASE, Web of Science, and PsycINFO (from inception onwards), without language/design restrictions, and apply backward citation screening. We shall hand-search the websites of 35 regulatory agencies/authorities, restricted publications from the Uppsala Monitoring Centre, and drug bulletins. Signals will be requested from the competent stakeholder, if absent from websites. We shall use VigiBase, the World Health Organization’s Global Individual Case Safety Report database, to determine the dates on which ADRs were reported. We shall manage records using EndNote (v. 8.2); one reviewer will screen titles/abstracts and full texts, a second will cross-validate the findings, and a third will arbitrate disagreements. Data will be charted via the Systematic Reviews Data Repository, following the same procedures as for data retrieval. Evidence will be categorized according to the Oxford Centre for Evidence-Based Medicine Levels of Evidence. Features of reports of ADRs will be coded. Tables will display frequencies of types of evidence and features of reports of ADRs. We shall use plots or pictograms (if appropriate) to represent the time from the first report of a suspected ADR to a signal.

**Discussion:**

We expect the findings from this review will allow a better understanding of global patterns of similarities or differences in terms of supporting evidence and timing of communications and identify relevant research questions for future systematic reviews.

**Systematic review registration:**

osf.io/a4xns

## Background

A systematic review of definitions, later adopted by the Council for International Organizations of Medical Sciences, defined a signal in pharmacovigilance as “information that arises from one or multiple sources (including observations and experiments), which suggests a new potentially causal association, or a new aspect of a known association, between an intervention and an event or set of related events, either adverse or beneficial, which would command regulatory, societal or clinical attention, and is judged to be of sufficient likelihood to justify verificatory and, when necessary, remedial actions” [1, 2].

Based on this definition, a signal can be supported by diverse levels of evidence, ranging from one or more reports of suspected adverse drug reactions (ADRs) to observational studies and randomized clinical trials [3–5]. On the other hand, signals of disproportionate reporting (SDRs) [1] can be highlighted solely by statistical associations resulting from ratios of observed versus expected numbers of reports (i.e. disproportionality) [6]. Different stakeholders can detect signals—including regulatory agencies, pharmaceutical companies, academics, independent research organizations, or patients and their carers—and avail themselves of several channels to disseminate them, such as peer-reviewed articles, abstracts, drug bulletins, websites, and restricted publications. Minutes of meetings of committees can also document discussions relating to signals [7].

Comparative assessments of signals discussed at the Pharmacovigilance Risk Assessment Committee have suggested that reports of ADRs often supported signals, but that support from multiple sources of evidence increased the likelihood of regulatory action (e.g. changes to Summaries of Product Characteristics) [8, 9]. Similar investigations carried out in the USA have highlighted the same finding [10, 11]. Regulatory actions, however, appear to be inconsistent across international settings; discrepancies in communications of the same risks of drug-induced harms in healthcare systems have been recorded, and it has been suggested that they may be better understood by characterizing the strength of the underpinning evidence [12, 13].

An important component of detection of signals of ADRs is clinical judgment of the data. The Bradford-Hill viewpoints or guidelines [14] and other structured methods of causality assessment [15] have typically guided clinical judgments in pharmacovigilance, but their intrinsic subjectivity has yielded low interrater agreement across independent assessors [16]. Though some methods have a higher interrater agreement than others, there appears to be no consensus on which ought to be the gold standard [17]. Consequently, regulators’ clinical judgments about features of reports of ADRs have occasionally been criticized by healthcare workers or their professional associations [18–21]. It has been reported that difficulties in assessing causality result in delays in the withdrawal of medicinal products from the market when fatal adverse events are attributed to their use [22]. Furthermore, the time intervals before signals of ADRs appear and regulatory actions follow are determined by the availability of information about the ADRs at the time of product launch; in fact, these intervals were shorter when ADRs were present in a sample of the Food and Drug Administration’s “drug label” at launch (e.g. severe liver injury with telithromycin or suicidal behaviour with varenicline) and longer when ADRs were absent [23]. Other accounts report that it may take 2–12 years for drug-induced harms to be communicated to healthcare workers after marketing authorization of a medicinal product [24–27]. These studies have computed the difference between dates of communication of signals and launch dates to measure the time-to-signal, while databases of reports of ADRs, such as VigiBase, could estimate this interval and account for unpublished records.

Over several decades of pharmacovigilance, many definitions of signals have been developed and applied, and different types of studies have been used to detect signals, resulting in a complex body of evidence whose breadth has been only partly characterized. Delays to withdrawals have been investigated systematically, while those relating to the time to signal have been limited in scope. Furthermore, while reports of ADRs remain a staple in signal detection, it is unclear to what extent their features (e.g. time to onset, dose-response relationship, positive de-/re-challenge) have been described or contributed to putative causal relationships. This is relevant when one considers that recent data-driven methods to prioritize signals arising from reports of ADRs are based on the quality of information of the reports themselves and a limited set of features, i.e. the presence of a narrative, the geographical distribution of the reports of ADRs, how recently reports of ADRs have been entered into a database, and a numerical measure of data completeness [28, 29]. Comprehensive overviews of the features of reports of ADRs that support signals may further inform improvements to such methods.

We therefore believe that it is worthwhile to conduct a scoping review, since such reviews are suitable for addressing broad exploratory questions pertaining to types of evidence in an area of research [30–32]

The aims of this scoping review are as follows: (a) to provide an evidence synthesis for decision-making at a regulatory level; (b) to collate signals currently in different platforms; (c) to produce an overview of the types and levels of evidence of studies that support signals; (d) to highlight any differences or similarities in assessment of the evidence used to support signals, with a particular focus on reports of ADRs; and (e) to better understand the delays before signals are detected and to clarify whether they have changed over time.

## Methods

The six-step framework introduced by Arksey and O’Malley [30] and refined by Levac et al. [31] and Colquhoun et al. [32] will set the structure of this protocol.

The review protocol has been registered in the Open Science Framework database (registration number: osf.io/a4xns) and is being reported in accordance with the reporting guidance provided in the Preferred Reporting Items for Systematic Reviews and Meta-Analyses Protocols (PRISMA-P) statement [33, 34] (see checklist in Additional file 1). The proposed scoping review will be reported in accordance with the reporting guidance provided in the Preferred Reporting Items for Systematic Reviews and Meta-analyses (PRISMA) extension for Scoping Reviews (PRISMA-ScR) [35].

### Step 1: Defining the research questions

We are aware of heterogeneous or inconsistent use of the term “signal” [36], that signals of ADRs may be communicated by various stakeholders (see below for definition), that over time signals have been supported by a wide range of types of evidence, and that pharmacovigilance regulation is context-dependent. Defining key concepts thus becomes essential, beginning with the elemental components of a signal: one or more medicinal product (see [37]) and one or more suspected adverse drug reaction (see [38]). Crucially, we understand the concept “signal of ADR”, its subtypes (i.e. indeterminate, verified, refuted), and that of “SDRs” as previously defined [1]. These definitions, however, would commit us to determine whether any form of evidence, even single reports of ADRs, would command attention (“whose?”) or regulatory action (“of what type?”); making such judgments for every published and unpublished record would result in an unmanageable amount of work. We further acknowledge the subjectivity that comes into play in assessing some evidence as signal and that our judgments may differ from those of study authors, introducing bias. We therefore decided to consider as signals any findings that are merely described as such (e.g. “these results should be considered as a signal” or “the association between “drug A” and “ADR B” is a signal”) without distinguishing subtypes: following-up signals to ascertain any subsequent verificatory action would entail substantial additional work. We consider SDRs, i.e. results of disproportionality analyses (e.g. proportional reporting ratio (PRR), information component), those that exceed thresholds for statistical significance set by the study authors [28, 39, 40].

Pharmacovigilance, as defined by the European Union directive 2010/84/EU [41], regulation 1235/2010 [42], Good Vigilance Practices – Module IX [7], and the International Council for Harmonisation of Technical Requirements for Pharmaceuticals for Human Use, efficacy guidelines E2A to E2F [43], will serve as references to contextualize pharmacovigilance in a global landscape.

We consider “stakeholders” to be healthcare practitioners and patients, national and international regulatory agencies or authorities, national and regional pharmacovigilance centres, non-governmental organizations, research institutions, academics, lawyers, and marketing authorization holders (i.e. pharmaceutical companies).

Based on these definitions, the questions this review will address are as follows:

1. What levels of evidence support signals of adverse drug reactions, and SDRs, communicated by drug regulatory authorities or other pharmacovigilance stakeholders?
2. What are the similarities and differences between the levels of evidence supporting signals from different stakeholders?
3. Have the levels of evidence changed over time?
4. What is the interval between a signal and the first report of the corresponding suspected adverse drug reaction?
5. Has this interval shortened or increased over the years?
6. In the case of signals supported by reports of adverse drug reactions, what features of the reports have led authors to signal suspected drug-related harms?

### Step 2: Identify relevant articles

To ensure that searches in electronic databases would retrieve articles relevant to the research questions, we consulted a medical librarian. We initially ran pilot searches based on keywords in the list of the included references of [1] and iteratively refined the queries in line with the published literature on search strategies for adverse effects [44, 45]. Moreover, since signals may be unpublished, we intend to carry out grey literature screening, which will involve hand searches of selected bulletins. The librarian provided suggestions on how to rigorously query grey literature search engines.

Relevant articles will be retrieved as follows:

We shall search MEDLINE (via PubMed, 1946–present), EMBASE (1974–present), PsycINFO (1806–present), and Web of Science (indexes SCI-EXPANDED, CPCI-S, 1945–present) for signals published by organizations involved in pharmacovigilance and/or independent research groups (e.g. regional pharmacovigilance centres or academic departments) and pharmaceutical companies. We shall use Web of Science to perform backward citation screening; if we cannot use Web of Science, we will use Google Scholar.

We shall search the English language websites of 35 regulatory authorities or organizations involved in the World Health Organization (WHO) Programme for International Drug Monitoring, using the websites’ search engines, to identify communicated signals, expanding the list of countries compiled by Onakpoya et al. [22] where possible. If signals are not publicly available in a country, we shall forward requests to the competent authority. If the evidence used to support signals is not publicly available, we shall issue freedom of information requests (where regulated) for assessment reports or equivalent documents.

We shall hand-search the Uppsala Monitoring Centre (UMC)’s SIGNAL Document, the WHO Pharmaceuticals Newsletter, the Full List of WHO Medical Product Alerts, the WHO Drug Information, Australian Prescriber, Prescrire International, and other drug bulletins available in English via the International Society of Drug Bulletins’ index for relevant records.

We shall query Google Scholar in incognito browser tabs. We shall evaluate the results as long as 20 results (two consecutive pages) yield at least one useful article.

We shall also search OpenGrey and GreyNet International.

We shall use VigiBase [46], to obtain additional information on the year in which suspected ADRs were first reported.

The planned data lock point for the retrieval of all records is the end of August 2020.

A draft search strategy for PubMed and Google Scholar and sources of grey literature are provided as Additional file 2.

### Step 3: Study selection

Since one of our goals is to better understand the evidence underpinning signals, we want to be as comprehensive as possible, while being able to remove irrelevant publications. Thus, we will not constrain by study design, language, or specific population: primary or secondary research using qualitative or quantitative methods will be eligible. We shall consider as eligible a priori disproportionality analyses [28, 39, 40], as these are exclusive to the detection of associations between medicinal products and ADRs.

Clearly, these criteria are insufficient for discarding all irrelevant publications, and we may obtain a large list of references. We shall therefore stipulate that any retrieved study should concern patients (i.e. excluding simulations and animal studies). We have therefore devised criteria for exclusion. First, descriptive, experimental, and observational study designs that are not unequivocally conducted to detect signals, even though the original authors may have described their findings as “signals”. An explicit description of the findings as “signals” in the abstract and in the full text will be necessary for eligibility. Secondly, different thresholds for significance may be used in disproportionality analyses, e.g. PRR ≥ 2 with Σ^2^ ≥ 4 with or without a minimum of three reports of ADRs. To account for this, we shall not apply standard thresholds, but we will require the presence of a threshold for significance, whether referenced or in the full text.

These criteria preserve the views of the original authors, minimize biases at our end, and allow us to work with a manageable data set.

#### Eligibility criteria

##### *Inclusion criteria*

1. Disproportionality analyses [28, 39, 40] that concern one or more medicinal products and one ADR or more and report at least one statistically significant association as per the threshold for significance adopted by the study authors.
2. Systematic reviews with or without meta-analyses, randomized controlled trials, observational studies, or more recent methods for signal detection (e.g. self-controlled case series, active surveillance) as detailed in [39], in which the original authors explicitly described any detected associations as “signal” in the abstract or full text.
3. Qualitative appraisals of individual reports or series of reports of ADRs, by either structured or unstructured methods (e.g. Naranjo’s algorithm, global introspection, and other methods for causality assessment [17]), that have been indexed as “signals” (e.g. signals from the UMC) or in which the original authors explicitly described one or more findings as “signal” in the abstract or full text.
4. Government or agency reports, working papers, and drug bulletins, whose entries have been indexed as signals or which explicitly describe an association between one or more medicinal products/ADRs as “signal” (viz. European Medicines Agency [47]).

Documents in languages other than English will be included if the review team can translate them into English. Requests for translations will be issued to the publishing stakeholder if the review team cannot translate.

##### *Exclusion criteria*

1. Abstracts or papers for which the full texts are unavailable.
2. Documents that claim that the evidence does not support a signal or that no signal was detected.
3. Signals that are communicated in the same or similar form or substance more than once by the same stakeholder (only the first signal in chronological order will be considered, if no new information appears in subsequent reports).
4. Non-English documents and websites for which a translation is unavailable.

#### Selection process

All retrieved records will be imported into EndNote™ X8.2. We shall deduplicate records manually, based on the lists of authors, titles, abstracts, journal, and pagination.

Retrieved titles and abstracts will be reviewed by DS (first author) against the inclusion and exclusion criteria. IJO (last author) will cross-validate the findings. Disagreements will be resolved by arbitration by JKA (second author).

### Step 4: Charting the data

Eligible records will be reviewed by full text by DS. IJO will cross-validate the findings. Disagreements will be resolved by arbitration by JKA. Data charting will follow the same process.

#### Descriptive statistics

We shall extract data to compute descriptive statistics in a form created in the Systematic Review Data Repository, adapted to scoping reviews [48]. Specifically, the tabs we shall use are “Publications” and “Design”. Data to be extracted will be publication year, country of origin, stakeholder, study design, type of evidence, metric for detection (in the case of SDRs), population involved, setting (or database for SDRs), eligibility criteria, possible confounding factors, brand name of the medicinal product(s) (where available) or active pharmaceutical ingredient(s), formulation, considered ADR(s), definition of signal, year of the case report of the suspected ADR that was first transmitted to VigiBase and that relates to the same medicinal product, and ADR in a considered signal and/or of occurrence of the ADR (fields: “FirstDateDatabase” or, if complete, “ReactionStartDate”).

#### Features of reports of ADRs

To extract the features of reports of ADRs that were considered as supporting putative causal relationships, we shall include a set of pre-determined codes in the extraction form as multiple-choice checkboxes, each followed by a free-text field. These are “Time-to-onset”, “Dose-response relationship”, “Positive de-challenge”, “Positive re-challenge”, “Site-specificity of ADR”, and “Population-specificity of ADR”. Quotations from the full texts will be inserted in the respective free-text field. DS has been involved in qualitative assessments of reports of ADRs, and the codes have been based on his experience.

The extraction form will include a free-text field “Other” to accommodate the inclusion of other unanticipated features of reports of ADRs supporting putative causal relationships. Quotations from the full texts will be inserted in the field when they do not fall in any of the pre-specified codes and will be iteratively assigned codes in the form itself.

### Step 5: Collating, summarizing, and reporting the results

We shall adopt a narrative approach to present the findings; these will be summarized by descriptive statistics and graphical illustrations after having standardized terminologies across included studies (medicinal products and ADRs) and codes that relate to the unanticipated features of reports of ADRs.

#### Standardization of terminologies

We shall code medicinal products to substance level, using the WHO Drug Dictionaries (WHO-DD, latest version available at the start of the review), and, where possible, code adverse events at the Low-Level Term level of the hierarchy in the Medical Dictionary for Regulatory Activities® (MedDRA) (latest version available at the start of the review), (e.g. “increased mortality” and seriousness of adverse events cannot be coded in MedDRA).

We shall translate types of evidence to the Oxford Centre for Evidence-Based Medicine levels of evidence framework

Codes extracted from the form in step 4 will be harmonized in the case of variance.

#### Descriptive statistics

We shall calculate absolute frequencies of types of evidence in Microsoft Excel and group them by type of stakeholder and/or year of publication. Similarly, we shall calculate the absolute frequencies of codes and publishing stakeholders.

#### Calculation of the time to signal

The complete (dd-mm-yy) date on which an ADR occurred, as stated in reports of ADRs entered in VigiBase, will be used to calculate the time-to-signal, by accounting for the date on which the signal relating to the same combination of medicinal product and the adverse effect was communicated. If the date of occurrence of the ADR is unavailable or incomplete, we shall use the first date on which the report was entered into VigiBase instead (field: UMCCalculated FirstDateDatabase). This field is necessarily complete, and follow-up reports are reconciled to it in VigiBase. For signals communicated by multiple stakeholders, the earliest date of communication will be considered. Depending on available resources, we may calculate the time-to-signal, beginning from the date on which cumulative disproportionality reaches statistical significance for the information component (lower limit of the 95% CI > 0).

#### Study findings and dissemination

##### Data synthesis

We shall present frequencies of types of evidence grouped by stakeholders in tables. We shall use figures (e.g. histograms) to present changes in frequencies of types of evidence over intervals of time.

We shall present all signals in tabular format, as combinations of medicinal product/ADR, or as medicinal product/medicinal product/ADR in the case of drug-drug interactions, together with their year of publication, stakeholder, eligibility criteria, population, setting, confounding factors, year of first entry in VigiBase, year of occurrence of the ADR (where available), and definition of signal. When signals are supported by spontaneous reports of ADRs, the codes of features of the reports of the ADRs will be included. Each signal will be accompanied by the time to signal.

A separate table will include SDRs and will include the same fields as above, plus the metric for detection (the definition of signal will be replaced by the specified threshold for significance and the database will replace the field “setting”).

We shall illustrate the time to signal for each stakeholder, in timeline plots and pictures (if appropriate).

Illustrations will be created using Microsoft Excel.

##### Dissemination

The results of the scoping review will consist of an overview of the levels of evidence underpinning signals. We shall highlight any discrepancies across stakeholders in levels of evidence, times to signal, and the most frequent features of reports of ADRs used to support putative causal relationships. We shall contextualize our findings with other published records and report the limitations of the study and recommendations for research. Variations from the published protocol will be presented and amendments made to the registered version. The full review will be submitted to a peer-reviewed journal for publication.

## Discussion

To the best of the authors’ knowledge, this would be the first study to systematically chart the evidence in support of signals and draw international comparisons of its levels using a standardized framework. Previous characterizations of the evidence underpinning “initial safety signals” [11] have focussed, for example, on Drug Safety Communications published by the US Food and Drug Administration [49]. These postings concern “important drug safety issue(s)” with “potential to alter the benefit–risk analysis for a drug in such a way as to affect decisions about prescribing or taking the drug” [50]. The agency’s website, however, also lists “potential signals of serious risks” [51], that concerns “about an excess of adverse events compared to what would be expected to be associated with a product’s use” [52]. This indicates that resorting to a pre-established set of webpages may result in the omission of relevant records and may misrepresent the activities of those regulatory agencies who refer to “signal” using different expressions. The time from the first report of an ADR to the communication of a related signal will also be quantified and compared across different healthcare settings, accounting for unpublished reports of ADRs available in VigiBase—the largest database of this kind. The findings from this review will underscore research questions for future reviews.

We expect to retrieve publications that report large lists of SDRs. This will pose some challenges, particularly during data charting, as the electronic form we choose may be unsuitable to accommodate all of them. Should this be the case, we shall adopt a customized Microsoft Excel sheet.

We anticipate that when abstracts do not transparently report methods, we may discard relevant records. In fact, there are no guidelines for reporting signals or SDRs. Furthermore, our exclusion criteria may result in the omission of eligible records: considering as eligible only records whose findings are described as “signals” is an approach that is insensitive to the use of other expressions (e.g. “safety risk”). In principle, contact with original authors would disambiguate those cases in which other expressions have been used, but might render such a broad review infeasible. In the case of contact with regulatory agencies, however, we may ascertain whether communications of, for example, “safety concerns” or “important safety concerns” were considered signals, as long as they reply to our queries. It is possible that we may not receive replies.

Furthermore, some publications may use discontinued terminologies to describe ADRs, such as the Coding Symbols for a Thesaurus of Adverse Reaction Terms, which have not been officially reconciled with MedDRA. This may introduce discrepancies in coding terms across terminologies. This is less likely to occur for medicinal products, as WHO-DD are quite comprehensive. However, WHO-DD are insensitive to vaccines; for example, trivalent inactivated influenza vaccine would be coded to “influenza vaccine”. We therefore plan to make this clear in the limitations of the resulting publication. Finally, as concerns the time to signal, the date of entry in VigiBase may be later than the date of receipt of a report to a national pharmacovigilance centre, which may underestimate the time to signal. Between the occurrence of an ADR and the date of entry in VigiBase, there may be a gap ranging from months to several years, thus exaggerating the time to signal. In the interest of transparency, we shall report both dates for each retrieved signal. Also, ADRs may have never been reported to VigiBase in association with some medicinal products, which would impede calculations of the time to signal.

## Supplementary information

**Additional file 1. **PRISMA-P 2015 Checklist

**Additional file 2. **Search strategy

## Data Availability

The data extraction form with the extracted data will be made public via osf.io. The full URL will be made available once the scoping review is published. Data used to calculate the time-to-signal will also be made available, since it will be included in the extraction form; the dates of onset of an ADR, or dates of reporting to VigiBase, will be available on request to the corresponding author.

## Abbreviations

ADR(s): Adverse drug reaction(s)
MedDRA: Medical Dictionary for Regulatory Activities®
PRR: Proportional reporting ratio
SDR(s): Signal(s) of disproportionate reporting
UMC: Uppsala Monitoring Centre
WHO: World Health Organization
WHO-DD: WHO Drug Dictionaries
