# Supplementary material for: Signals of adverse drug reactions communicated by pharmacovigilance stakeholders: protocol for a scoping review of the global literature
Source: Syst Rev. 2020 Aug 13;9:180. doi: 10.1186/s13643-020-01429-z (PMC7425142; doi:10.1186/s13643-020-01429-z)
Supplement: Supplementary file 2 — Additional file 2. Search strategy [file 13643_2020_1429_MOESM2_ESM.pdf]

Supplementary Materials

Search strategy

The search strategy reported below refers to PubMed and has been converted to be run in the other prespecified databases.

Electronic database (PubMed)

(“pharmacovigilance”[tiab] OR “Product Surveillance, Postmarketing”[mh] OR “Adverse Drug Reaction Reporting Systems”[mh] OR “Adverse event reporting system”[tiab] OR surveillance[ti] OR “safety surveillance”[tiab] OR “post-marketing surveillance”[tiab] OR “postmarketing surveillance”[tiab] OR “drug surveillance”[tiab] OR "surveillance system"[tiab] OR "surveillance database"[tiab] OR "reporting system"[tiab] OR "reporting database"[tiab])

AND

(”adr”[tiab] or ”adrs”[tiab] or adverse event*[tiab] OR associat*[tiab] OR adverse drug event*[tiab] OR adverse drug reaction*[tiab] OR adverse drug effect*[tiab] OR adverse effect*[tiab] OR side effect*[tiab] OR undesirable effect*[tiab] OR "drug-induced"[tiab] OR "drug induced"[tiab] OR "drug-related"[tiab] OR "drug related"[tiab] OR "treatment-emergent"[tiab] OR “drug safety”[tiab] OR “product safety”[tiab] OR "patient safety"[tiab] OR "medicine safety"[tiab] OR safety issue*[tiab] OR safety problem*[tiab] OR safety concern*[tiab] OR ”tolerability"[tiab] OR ”toxicity"[tiab] OR unexpected effect*[tiab] OR unexpected event*[tiab] OR ”safety”[tiab] OR ”safe”[tiab] OR complication*[tiab] OR “Chemically Induced”[sh] OR “adverse effects”[sh] OR ”drug effects”[sh] OR

”complications”[sh] OR "drug interactions"[mh] OR “Drug-Related Side Effects and Adverse Reactions”[mh])

AND

("pharmacovigilance analysis"[tiab] OR signal*[tiab] OR "signal detection"[tiab] OR alert*[tiab] OR trigger*[tiab] OR spontaneous report*[tiab] OR spontaneously report*[tiab] OR event report*[tiab] OR safety report*[tiab] OR ” Unknown ADR”[tiab] OR ” Unknown ADRs”[tiab])

NOT

(“retracted publication”[pt] OR “Editorial”[pt] OR “Comment”[pt] OR “Letter”[pt] OR “News”[pt] OR “Surveys and Questionnaires”[majr] OR “Signal Transduction”[majr] OR "Published Erratum"[pt])

Grey literature: Google Scholar

(“signal” OR “signals”) AND (“side effect” OR “ADR” OR “adverse drug reaction” OR “adverse event”) AND “pharmacovigilance”

Grey literature: signals publicly available on regulatory websites

List of English language websites of drug regulatory agencies drawn from Onakpoya et al. [1] adapted for searches of signals of adverse drug reactions with medicinal products.

Numbered items followed by a letter were added by DS after consultation with Uppsala Monitoring Centre's Global Services and represent additional members of the WHO Programme for International Drug Monitoring.

1. ***Australia:*** Therapeutic Goods Administration (TGA): https://www.tga.gov.au/all-alerts#summary-s

2. Bahamas: Bahamas National Drug Agency (BNDA): http://www.corp.phabahamas.org/hospitals-services/bnda/

3. ***Bhutan:*** Drug Regulatory Authority: http://dra.gov.bt/

4. ***Botswana:*** Ministry of Health, Republic of Botswana: http://www.moh.gov.bw/ (no Medicines Regulatory Authority)

5. ***Canada:*** Health Canada Drug Product Database (DPD): http://www.hc-sc.gc.ca/dhp-mps/prodpharma/databasdon/index-eng.php

6. ***European Union:*** European Medicines Agency (EMA): http://www.ema.europa.eu/ema/

7. ***Gambia:*** Medicine Regulatory Authority: http://www.mca.gm/

8. ***Ghana:*** Ghana Food and Drugs Authority (FDA): http://www.fdaghana.gov.gh/

8a**. *Hong Kong***: Hong-Kong's Department of Health Drug Office http://www.drugoffice.gov.hk/eps/do/en/level.html

9. ***India:*** Central Drugs Standard Control Organization (CDSCO): http://www.cdsco.nic.in/forms/Default.aspx

10. ***Jamaica:*** Ministry of Health, Kingston, Jamaica: http://moh.gov.jm/?s=drug+regulation

10a. ***Japan***'s Pharmaceuticals and Medical Devices Authority:
https://www.pmda.go.jp/english/

11. ***Kenya:*** Pharmacy and Poisons Board, Kenya: http://pharmacyboardkenya.org/

11a. ***Korea (Republic of)***: Korea Institute of Drug Safety and Risk Management
https://www.drugsafe.or.kr/en/index.do

12. ***Liberia:*** Liberian Medicines Health Products Regulatory Authority:
https://healthresearchweb.org/en/liberia/research_regulation_4284

12a ***Malaysia***: National Pharmaceutical Regulatory Agency
https://www.npra.gov.my/index.php/en/

13. ***Malta:*** Malta Medicines Authority: http://www.medicinesauthority.gov.mt/home?l=1

14. ***Namibia:*** Namibia Medicines Regulatory Council: http://www.nmrc.com.na/

15. ***New Zealand:*** New Zealand Medicines and Medical Devices Safety Authority:
http://www.medsafe.govt.nz/index.asp

16. ***Nigeria:*** National Agency for Food and Drug Administration and Control (NAFDAC):
http://www.nafdac.gov.ng/

17. ***Pakistan:*** Drug Regulatory Authority of Pakistan (DRAP):
http://www.dra.gov.pk/gop/index.php?q=aHR0cDovLzE5Mi4xNjguNzAuMTM2L2RyYXAv

18. ***Philippines:*** Food and Drug Administration, Philippines (FDA): https://www.fda.gov.ph

19. ***Rwanda:*** Ministry of Health, Republic of Botswana:
http://www.moh.gov.rw/index.php?id=2 (no Medicines Regulatory Authority)

19a. ***Saudi Arabia:*** Saudi Food and Drug Administration https://www.sfda.gov.sa/En/Pages/default.aspx

20. ***Sierra Leone:*** Pharmacy Board of Sierra Leone: http://www.pharmacyboard.gov.sl/

21. ***Singapore:*** Health Sciences Authority: http://www.hsa.gov.sg/content/hsa/en.html

22. ***South Africa:*** South Africa Medicines Control Council (MCC): http://www.mccza.com/

23. ***Sri Lanka:*** Cosmetics, Devices and Drug Regulatory Authority: http://www.cdda.gov.lk/

24. ***Tanzania:*** Tanzania Food and Drugs Authority (TFDA): http://www.tfda.or.tz/

25. ***Thailand:*** Food and Drug Administration, Thailand: http://www.fda.moph.go.th/eng/index.stm

26. ***Trinidad and Tobago:*** Ministry of Health, Trinidad and Tobago: http://www.health.gov.tt/sitepages/default.aspx?id=93

27. ***Uganda:*** National Drug Authority: http://www.nda.or.ug/

28. ***United Kingdom:*** Medicines and Healthcare products Regulatory Agency (MHRA): https://www.gov.uk/government/organisations/medicines-and-healthcare-products-regulatory-agency

29. ***USA:*** Food and Drug Administration (FDA): http://www.fda.gov/

30. ***Zambia:*** Pharmaceutical Regulatory Authority (PRA): http://www.zamra.co/

31. ***Zimbabwe:*** Medicines Control Authority of Zimbabwe (MCAZ): http://www.mcaz.co.zw/

References

1. Onakpoya IJ, Heneghan CJ, Aronson JK. Post-marketing withdrawal of anti-obesity medicinal products because of adverse drug reactions: a systematic review. BMC Medicine. 2016;14(1):191.
